# Two-Phase Occupational Screening in Coal-Fired Power Plant Workers: Anamnestic Risk Stratification and Targeted Low-Dose CT Findings

**DOI:** 10.3390/diagnostics16193202

**Published:** 2026-10-02

**Authors:** Adrian Cranta, Eugen Osiac, Dan Ionuț Gheonea, Costin Teodor Streba, Carmen Nicoleta Oancea, Ioana Andreea Gheonea, Madalin Niculici, Cristina Ciofiac, Daiana Petrescu, Mircea-Sebastian Șerbănescu

**Affiliations:** 1 Biophysics Department, University of Medicine and Pharmacy of Craiova, 2 Petru Rareș Street, 200349 Craiova, Romania; adrian.cranta@umfcv.ro (A.C.); eugen.osiac@umfcv.ro (E.O.); 2 Centre for Research in Gastroenterology and Hepatology (CCGH), University of Medicine and Pharmacy of Craiova, 200349 Craiova, Romania; dan.gheonea@umfcv.ro (D.I.G.); madalin.niculici@umfcv.ro (M.N.); 3 Gastroenterology Department, University of Medicine and Pharmacy of Craiova, 2 Petru Rareș Street, 200349 Craiova, Romania; 4 Pneumology Department, University of Medicine and Pharmacy of Craiova, 2 Petru Rareș Street, 200349 Craiova, Romania; costin.streba@umfcv.ro; 5 Biochemistry Department, University of Medicine and Pharmacy of Craiova, 2 Petru Rareș Street, 200349 Craiova, Romania; carmen.oancea@umfcv.ro; 6 Department for Community Projects, University of Medicine and Pharmacy of Craiova, 2 Petru Rareș Street, 200349 Craiova, Romania; cristina.ciofiac@umfcv.ro; 7 Radiology and Imaging Department, University of Medicine and Pharmacy of Craiova, 2 Petru Rareș Street, 200349 Craiova, Romania; ioana.gheonea@umfcv.ro; 8 Informatics and Biostatistics, University of Medicine and Pharmacy of Craiova, 2 Petru Rareș Street, 200349 Craiova, Romania; mircea.serbanescu@umfcv.ro

**Keywords:** occupational health, coal-fired power plant, low-dose CT, lung cancer screening, Lung-RADS, emphysema, coronary artery calcium

## Abstract

**Background:** Employees at coal-fired power plants experience cumulative occupational exposure to respirable particulates alongside a high prevalence of smoking, both contributing to chronic respiratory disease and elevated lung cancer risk, and organized-screening implementation data remain scarce in this specific industrial setting outside asbestos-exposed cohorts. We evaluated a two-step occupational screening strategy integrating an anamnestic questionnaire with targeted low-dose CT (LDCT) imaging, assessing risk distribution, structured imaging findings, and the discriminative value of questionnaire-based triage relative to its underlying continuous predictors. **Methods:** In this cross-sectional study, all workers at a coal-fired power plant completed a structured questionnaire (F1) and were classified as high-risk or non-high-risk using a predefined eligibility rule (respiratory symptoms, or age ≥ 50 years with current/former smoking). All workers were invited to voluntary LDCT (F2), interpreted using Lung-RADS v2022 with structured emphysema and coronary artery calcium (CAC) assessment. Associations were assessed using Fisher’s exact tests, multivariable logistic regression, and ROC analysis with DeLong’s test; voluntary LDCT uptake was characterized with a multivariable propensity model. **Results:** Among 870 screened workers, 364 (41.8%) were high-risk. LDCT uptake was higher among high-risk workers (130/364, 35.7%) than non-high-risk workers (56/506, 11.1%), yielding 186 examinations. Only 14/186 (7.5%) had Lung-RADS ≥ 3. High-risk status was associated with higher prevalence of any nodule (80.8% vs. 62.5%; OR 2.52) and CAC (46.9% vs. 14.3%; OR 5.30); a composite abnormality outcome was present in 92.3% vs. 69.6% (OR 5.23). Only age (OR 1.11/year) and ever-smoking status (OR 4.38) independently predicted abnormalities on multivariable analysis; dichotomized risk classification showed reduced discrimination for CAC versus a continuous age–smoking model (AUC 0.647 vs. 0.779). **Conclusions:** Questionnaire-based triage effectively enriched the LDCT-screened population for smoking-related and cardiometabolic risk. Age and smoking history, rather than the full questionnaire, principally drove imaging abnormalities, supporting an integrated occupational model combining lung cancer screening with COPD and cardiovascular risk management.

## 1. Introduction

Coal-based power generation involves the combustion of coal, a process that generates airborne particulate matter, including fine particulates, coal dust, and fly ash, together with gaseous pollutants. It is worth distinguishing two overlapping but compositionally distinct exposure profiles in the literature: coal-mine dust (relevant to extraction) and combustion-derived fly ash (relevant to power generation). In coal-mine-dust-exposed cohorts, an extensive literature links cumulative exposure to interstitial lung disease [1,2] and to elevated mortality from chronic obstructive pulmonary disease (COPD), independent of smoking status [3]. Among fly-ash and combustion particulate-exposed workers specifically, occupational exposure has been associated with chronic respiratory symptoms such as cough, phlegm, wheezing, and dyspnea that are potentially mediated through oxidative stress and genotoxic effects of inhaled heavy metals and fine particulates [4]. Adults residing near coal-fired power plants likewise show significantly elevated rates of respiratory symptoms compared with unexposed populations [5]. Employees in these environments may experience chronic occupational exposure, which adds to background environmental exposure and lifestyle-related factors such as smoking. The interaction of these factors may promote the development of obstructive respiratory diseases and, in high-risk individuals, may increase the likelihood of lung cancer.

Low-dose computed tomography (LDCT) has been shown in two landmark randomized controlled trials to reduce lung cancer mortality among high-risk current and former smokers: the U.S. National Lung Screening Trial (NLST) demonstrated an approximately 20% relative reduction in lung cancer mortality with LDCT compared with chest radiography [6], and the Dutch–Belgian NELSON trial subsequently confirmed a comparable, and in some subgroups larger, mortality benefit using volumetric CT [7]. These trials have established organized LDCT screening as standard practice in defined high-risk populations; however, screening criteria, high false-positive rates, and the resulting burden on healthcare resources remain unresolved challenges in broader implementation [8]. Extending its use beyond clearly defined high-risk populations can result in more false-positive findings and increased demand on healthcare resources. Consequently, practical screening programs often include a pre-screening step to identify individuals most likely to benefit from LDCT and to organize follow-up pathways.

Anamnestic questionnaires allow for the integrated clinical review of multiple risk factors, including age, smoking exposure (in terms of intensity and duration), environmental pollution, occupational factors, respiratory symptoms, and family history. This approach parallels, in simplified form, the logic of formal lung-cancer risk prediction models: PLCOm2012, for example, incorporates a broader panel of risk factors beyond age and cumulative smoking pack-years and has consistently shown improved sensitivity and positive predictive value relative to simple binary eligibility criteria such as NLST-style age/pack-year thresholds [9]. Across validated risk prediction models more generally, discrimination for future lung cancer has ranged from an AUC of approximately 0.61 to 0.81 depending on the model and validation cohort, with these models consistently outperforming simple criteria-based eligibility in sensitivity and specificity [10]. When implemented at a population scale in professional settings, such tools can provide a structured triage system and simultaneously identify modifiable risk factors, such as active smoking, ongoing harmful exposures, or the need for medical assessment in the presence of symptoms.

Beyond nodule detection with Lung-RADS classification, LDCT scans routinely reveal clinically relevant incidental findings unrelated to lung cancer [11]. The most frequently reported significant incidental findings were emphysema and coronary artery calcification [12,13], underscoring that a well-designed occupational LDCT screening program can serve as a broader cardiopulmonary health surveillance tool for the screened workforce, and not solely as a cancer-detection instrument. Despite repeated calls for the development of well-organized lung cancer screening programs in occupationally exposed populations [14], published implementation data from industrial workforces outside asbestos-exposed cohorts remain limited, and coal-fired power generation, a setting combining combustion-derived particulate exposure with typically high background smoking prevalence, remains particularly underrepresented in this literature.

This study evaluates a two-step pulmonary screening campaign conducted in a coal-fired power plant. The first step was an anamnestic questionnaire (F1), which was used to classify workers according to individual risk. The second step offered LDCT (F2) to all workers regardless of risk category, with participation on a voluntary basis, and results reported in a standardized manner according to Lung-RADS v2022 [15]. The study aimed to (1) describe the distribution of anamnestic risk in this workforce; (2) characterize LDCT findings, including Lung-RADS classification, the presence of emphysema, and coronary artery calcium (CAC), as structured indicators of both oncologic and broader cardiopulmonary health; and (3) assess the integration between questionnaire risk and LDCT outcomes to optimize occupational health interventions.

## 2. Materials and Methods

### 2.1. Study Design and Setting

This was a cross-sectional screening campaign conducted among workers employed at a single coal-fired power plant. The dataset includes two linked components: anamnestic screening (F1) and LDCT imaging (F2). Personal identifiers were masked in the exported dataset and were used only for deterministic linkage between F1 and F2 during analysis.

### 2.2. Participants

All workers who completed the anamnestic questionnaire during the screening period were included in the F1 analysis. Workers who subsequently underwent LDCT were included in the F2 analysis. All workers were invited to undergo LDCT regardless of anamnestic risk category. Uptake was voluntary, as shown in Section 3.2 and modeled explicitly in the statistical analysis below; participation was not simply a function of risk category but was independently associated with age, smoking status, respiratory symptoms, and occupational exposure. The F2 cohort should therefore be interpreted as a self-selected, enriched subset shaped by multiple correlated factors, not a random sample of the workforce, and all subsequent association and discrimination analyses characterize this imaged subset rather than the full screened population.

### 2.3. Anamnestic Questionnaire

The questionnaire recorded gender and age, residence (urban/rural) and environmental pollution proxies (urban traffic; type of household heating and cooking fuels; proximity to an industrial pollution source), smoking status (active, former, passive, never) and quantitative smoking history (years smoked, years since quitting, cigarettes per day), occupational exposure to respiratory hazards (a single yes/no item, without quantitative detail on job category, duration, cumulative exposure, or use of protective equipment), family history suggestive of lung cancer, respiratory symptoms (e.g., persistent cough, recurrent infections, exertional dyspnea, hoarseness, hemoptysis, chest pain), prior thoracic imaging within the last 12 months and whether abnormal findings required follow-up, and prior history of malignancy.

### 2.4. Risk Classification

Participants were classified as high-risk if they reported respiratory symptoms, or if age ≥ 50 years co-occurred with current or former smoking; all other participants were classified as non-high-risk. This binary rule was designed to align questionnaire-based triage with LDCT screening eligibility principles used in international guidelines [16]. Full derivation and per-subject classification for all 870 participants are provided in Supplementary Material S1. Under this classification, 364/870 participants (41.8%) were high-risk and 506/870 (58.2%) were non-high-risk. Among the 364 high-risk participants, 175 (48.1%) met the criterion through age ≥ 50 years with current or former smoking only, 112 (30.8%) through respiratory symptoms only, and 77 (21.2%) through both criteria simultaneously.

### 2.5. LDCT Acquisition

Non-contrast chest LDCT was performed on a single multidetector CT scanner (Biograph 20; Siemens Healthineers, Erlangen, Germany), a hybrid PET/CT system with a 20-slice diagnostic multidetector CT gantry) for all 186 participants throughout the screening campaign. Acquisition slice thickness was 1.5 mm. Two pulmonary reconstructions were generated for each examination, at 0.75 mm and 1 mm slice thickness, to account for variability in patient body habitus and to reduce artefacts in overweight participants. Tube voltage was fixed at 120 kVp (140 kVp for overweight participants) with a fixed tube current-time product of 35 mAs; automatic dose modulation was not used. Images were reconstructed using a pulmonary (sharp) kernel without iterative reconstruction. All participants were positioned supine and scanned in maximal inspiration according to a standardized protocol. CTDIvol values were extracted automatically from the DICOM header of each examination.

### 2.6. LDCT Interpretation

All examinations were interpreted by a single specialist radiologist (single-reader design). The reader holds certification in “EuCanScreen—Lung cancer screening with low-dose CT: a practical approach,” obtained at the National Korányi Institute of Pulmonology, Hungary. Nodule detection and measurement (diameter and volume) were performed using a commercially available artificial intelligence-based pulmonary nodule assessment system (Rayscape, Mindfully Technologies SRL, Timișoara, Romania; https://rayscape.ai, accessed between 25 October and 23 November 2025) which provided automated nodule detection and volumetric/diametric measurement; all AI-generated nodule diameters were subsequently re-measured manually by the radiologist to verify software accuracy.

### 2.7. Statistical Analysis

Descriptive statistics were used to summarize the F1 and F2 cohorts. LDCT findings were described using counts and percentages. Associations between the binary anamnestic risk classification and CT outcomes were assessed using Fisher’s exact test and odds ratios (OR), both for a composite outcome (any of: nodule, emphysema, or CAC) and, separately, for clinically actionable outcomes (Lung-RADS ≥ 3, moderate-to-severe CAC ≥ 1/3, moderate-to-severe emphysema), given the limited clinical specificity of the composite (Section 3.4). Multivariable logistic regression (composite outcome ~ age + smoking status + sex + occupational exposure + respiratory symptoms) was used to identify independent predictors; model diagnostics included events-per-parameter (EPV) and variance inflation factors (VIF) for collinearity, and a parsimonious two-predictor sensitivity model (age + smoking only) was additionally fitted for the actionable outcomes, which had too few events to support the full five-predictor model. Discriminative performance of a continuous age–smoking logistic model versus the binary risk classification was compared using receiver operating characteristic (ROC) curve analysis, with areas under the curve (AUC) compared using DeLong’s test for correlated ROC curves. Because LDCT uptake was voluntary, a multivariable propensity model for uptake (age, smoking status, symptoms, sex, occupational exposure) was fitted on the full F1 cohort to characterize the determinants of participation; inverse-probability weighting was not applied to the discriminative estimates, and the reported AUCs are unweighted. Analyses were performed in Python (v3.12; Python Software Foundation, Wilmington, DE, USA) using pandas (v3.0), SciPy (v1.17), statsmodels (v0.15), and scikit-learn (v1.8). Figures were generated using GraphPad Prism (v8; GraphPad Software, Boston, MA, USA).

**Figure 1:**
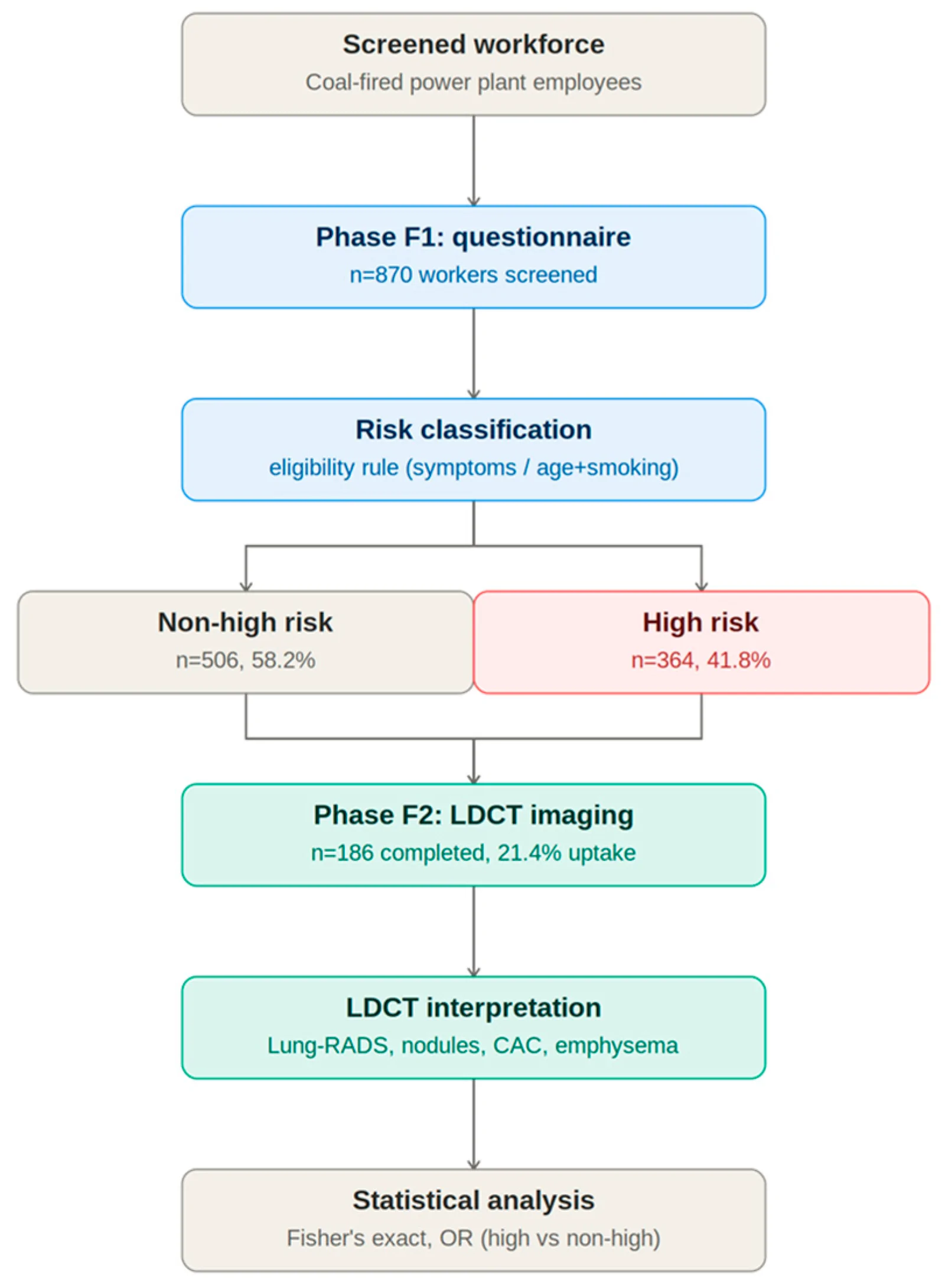
Flowchart of the screening program, from workforce enrollment through anamnestic risk stratification and low-dose CT (LDCT) imaging. All workers employed at the coal-fired power plant were invited to complete the anamnestic questionnaire (Phase F1; *n* = 870). Each participant was classified as high-risk or non-high-risk using the eligibility rule described in Methods (Supplementary Material S1). All workers were subsequently invited to undergo LDCT imaging (Phase F2) regardless of risk category; uptake was voluntary and varied substantially by risk group, with completed exams (*n* = 186) undergoing structured radiological interpretation (Lung-RADS v2022, nodule characteristics, coronary artery calcium, and emphysema grading) and integration with anamnestic risk in the statistical analysis.

The overall screening workflow, from workforce enrollment through anamnestic risk stratification and LDCT imaging, is summarized in Figure 1.

## 3. Results

### 3.1. Cohort and Anamnestic Risk Distribution

A total of 870 workers completed the anamnestic questionnaire (F1). Mean age was 49.3 ± 7.5 years and 628/870 (72.2%) were male. Of these, 409 (47.0%) had never smoked, 226 (26.0%) were current smokers, 202 (23.2%) were former smokers, and 33 (3.8%) reported passive smoking exposure only. Occupational exposure to respiratory hazards was reported by 94.5% of respondents (810/857; 13 missing). Respiratory symptoms were reported by 189/861 (22.0% of respondents) (Table 1).

### 3.2. LDCT Uptake, Its Determinants, and Dose Descriptors

LDCT screening (F2) was completed by 186 of the 870 workers (21.4%), with uptake markedly higher among high-risk workers: 130/364 (35.7%) versus 56/506 (11.1%) among non-high-risk workers. Among those who completed LDCT, the average age was 51.6 ± 5.9 years, and 132/186 (71.0%) were male. Regarding radiation dose, the mean CTDIvol was 1.92 ± 0.58 mGy, with a median of 1.87 mGy and an interquartile range (IQR) of 1.51–2.23.

Because participation was voluntary, a multivariable logistic model of LDCT uptake was fitted on the full F1 cohort (*n* = 870) to characterize its determinants beyond the binary risk category. Uptake was independently associated with age (OR 1.06 per year, 95% CI 1.03–1.09, *p* < 0.001), ever-smoking status (OR 6.51, 95% CI 4.28–9.89, *p* < 0.001), respiratory symptoms (OR 1.98, 95% CI 1.34–2.91, *p* = 0.001), and occupational exposure (OR 3.66, 95% CI 1.38–9.73, *p* = 0.009), with a borderline association for male sex (OR 0.69, 95% CI 0.45–1.03, *p* = 0.072). This confirms that participation reflected a combination of risk-related factors beyond the binary risk stratum alone (Table 2).

### 3.3. Structured LDCT Findings

**Nodules**

A pulmonary nodule was detected in 140 of the 186 participants (75.3%), with a median maximum diameter of 4.5 mm (IQR 2.7–6.0 mm). Among all nodules identified, most were solid (71.9%) or calcified (23.9%), while ground-glass and part-solid nodules were far less common, accounting for 3.7% and 0.6%, respectively.

**Lung-RADS**

**Figure 2:**
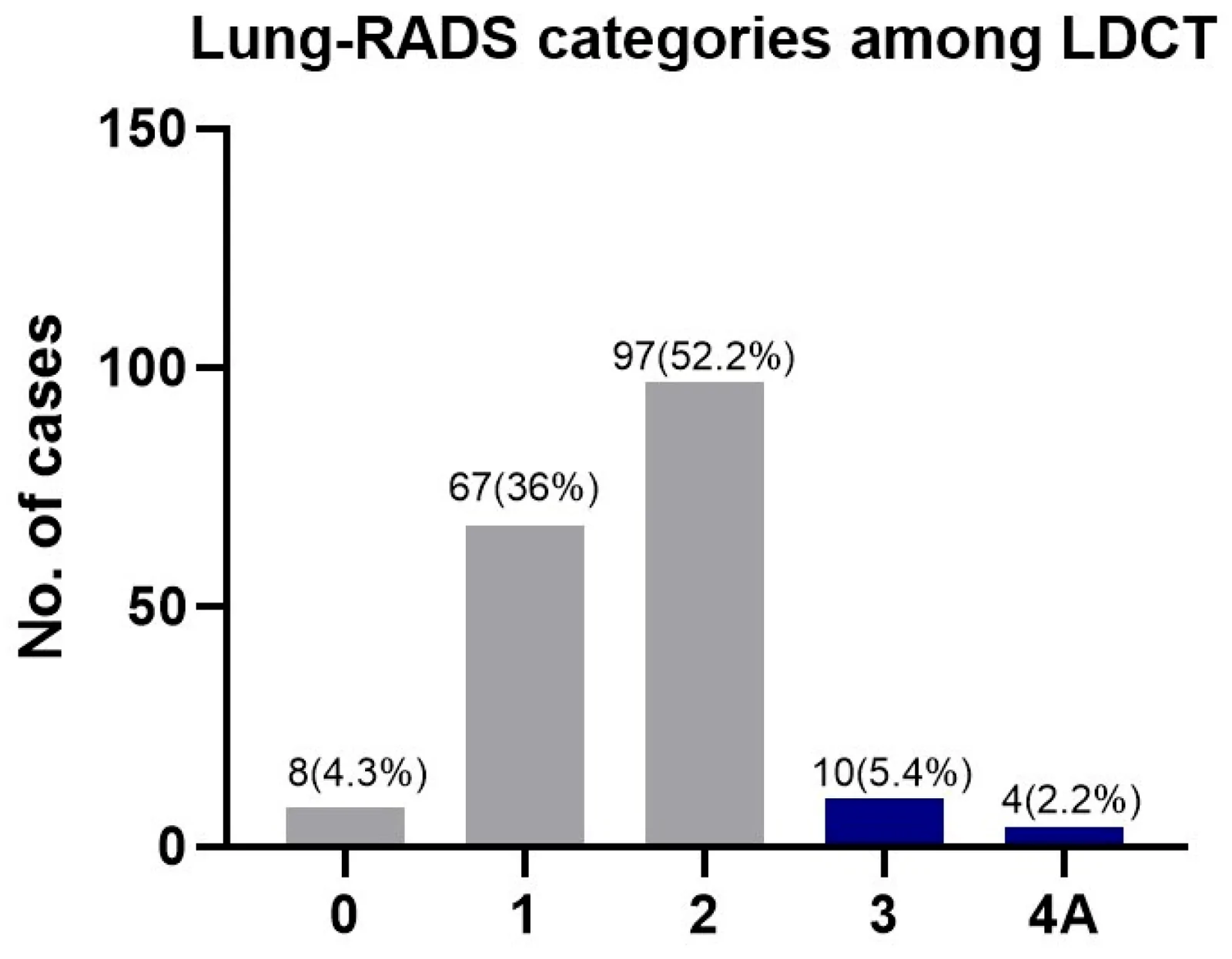
Distribution of Lung-RADS v2022 categories among LDCT examinations (N = 186). Category 0 (indeterminate, *n* = 8, 4.3%), category 1 (negative, *n* = 67, 36.0%), category 2 (benign, *n* = 97, 52.2%), category 3 (probably benign, *n* = 10, 5.4%), and category 4A (suspicious, *n* = 4, 2.2%). Categories 3 and 4A (highlighted in blue) together represent Lung-RADS ≥ 3 findings requiring closer follow-up (*n* = 14, 7.5% of the F2 cohort).

Participants were classified as Lung-RADS 0 in 8 cases (4.3%), category 1 in 67 (36.0%), category 2 in 97 (52.2%), category 3 in 10 (5.4%), and category 4A in 4 (2.2%). Overall, 14 participants (7.5%) fell into Lung-RADS category 3 or higher (Figure 2).

**Airways and parenchyma**

Emphysema was identified in 42 participants (22.6%), most often mild in severity (20.4%); moderate or confluent emphysema, the more clinically significant grades, was present in only 4 participants (2.2%). Based on free-text radiology reports, fibrotic or scar-like changes were noted in 18 cases (9.7%), pleural abnormalities in 10 (5.4%), airway mucus or bronchitis-type changes in 17 (9.1%), and infectious or inflammatory patterns in 12 (6.5%).

**Cardiovascular incidental findings**

**Figure 3:**
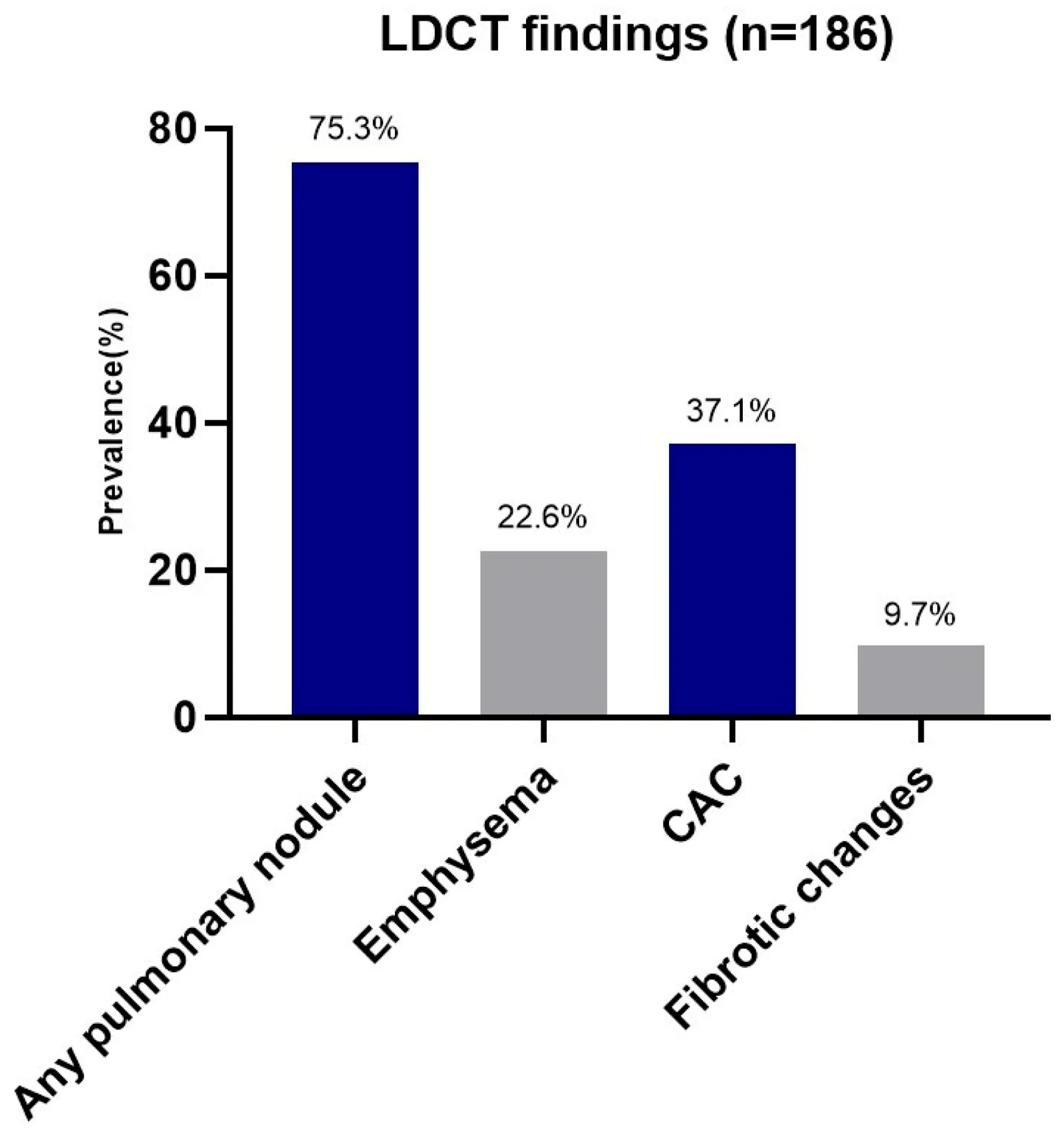
Prevalence of selected LDCT findings among the F2 cohort (*n* = 186): any pulmonary nodule (75.3%), coronary artery calcium/CAC (37.1%), emphysema (22.6%), and fibrotic changes on free-text review (9.7%).

Visually assessed coronary artery calcification was present in 69 participants (37.1%), most commonly at a mild level, involving less than one-third of the vessel (31.2%). Moderate-to-severe CAC, involving at least one-third of the vessel, the grade most consistently linked to clinically significant cardiovascular risk, was reported in 11 cases (5.9%) (Figure 3).

All key findings in the LDCT scan are reported in Table 3.

### 3.4. Integration of Anamnestic Risk and LDCT Finding

Using the binary anamnestic risk classification (high-risk, *n* = 130 vs. non-high-risk, *n* = 56, within the F2 subsample), high risk was associated with a significantly higher prevalence of any recorded nodule (80.8% vs. 62.5%; OR 2.52, Fisher’s *p* = 0.010, 95% CI 1.26–5.05) and a markedly higher prevalence of visual CAC (46.9% vs. 14.3%; OR 5.30, *p* < 0.0001, 95% CI 2.33–12.09). Emphysema prevalence was similar between groups (23.1% vs. 21.4%; OR 1.10, *p* = 0.851, 95% CI 0.52–2.35), and the proportion of Lung-RADS ≥3 findings was low and did not differ significantly (7.7% vs. 7.1%; OR 1.08, *p* = 1.000, 95% CI 0.32–3.61) (Table 4).

**Figure 4:**
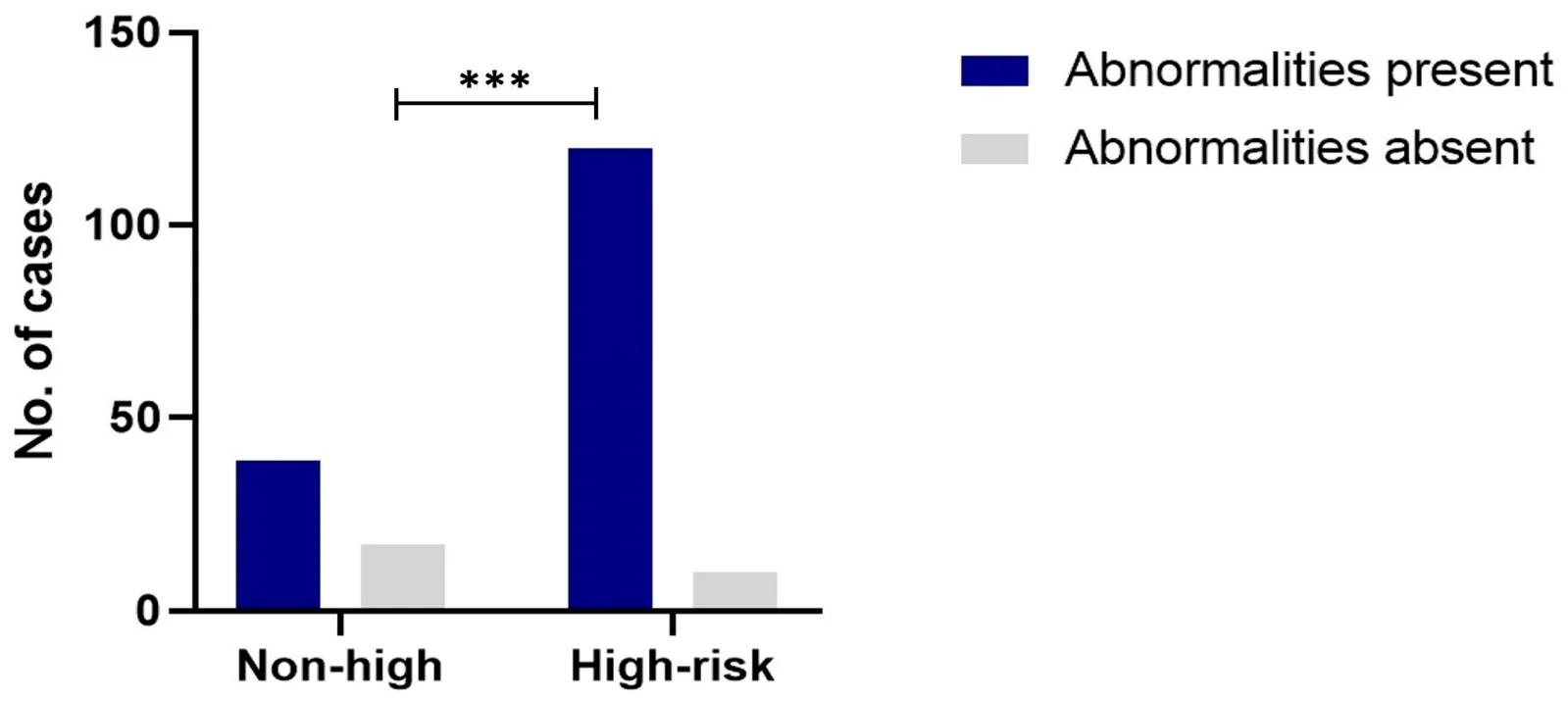
Number of participants with at least one LDCT abnormality present versus absent (composite outcome: any nodule, emphysema, or CAC), by anamnestic risk group. High-risk participants had significantly more abnormalities present than non-high-risk participants (120/130, 92.3% vs. 39/56, 69.6%; OR 5.23, *p* = 0.0002). *** the result is statistically significant with a *p*-value less than 0.001.

A composite outcome (any of nodule, emphysema, or CAC, at any severity) further sharpened this association: 92.3% of high-risk participants had at least one imaging abnormality, compared with 69.6% of non-high-risk participants (OR 5.23, Fisher’s *p* = 0.0002) (Figure 4). However, because small, incidental, low-specificity findings (in particular small solid or calcified nodules and mild CAC) dominate this composite, and because it was present in over 90% of high-risk participants, its clinical specificity is limited; this composite should not, by itself, be interpreted as evidence that the anamnestic strategy identifies workers likely to derive clinical benefit from LDCT.

To assess whether this association extended to clinically actionable disease, the same comparison was repeated restricting each finding to its clinically significant threshold: Lung-RADS ≥ 3 (rather than any nodule), moderate-to-severe CAC (≥1/3, rather than any CAC), and moderate-to-severe emphysema (rather than any emphysema). None of these actionable outcomes differed significantly between risk groups (Table 5), including CAC, for which the point estimate remained elevated (OR 4.58) but the association was no longer statistically significant (*p* = 0.178), reflecting the small number of moderate-to-severe cases (11 in the whole F2 cohort). This indicates that the strong composite association reported above is driven predominantly by common, low-specificity findings rather than by disease meeting conventional thresholds for clinical action, and tempers the interpretation of the anamnestic classification as a tool for identifying workers likely to require intervention.

**Figure 5:**
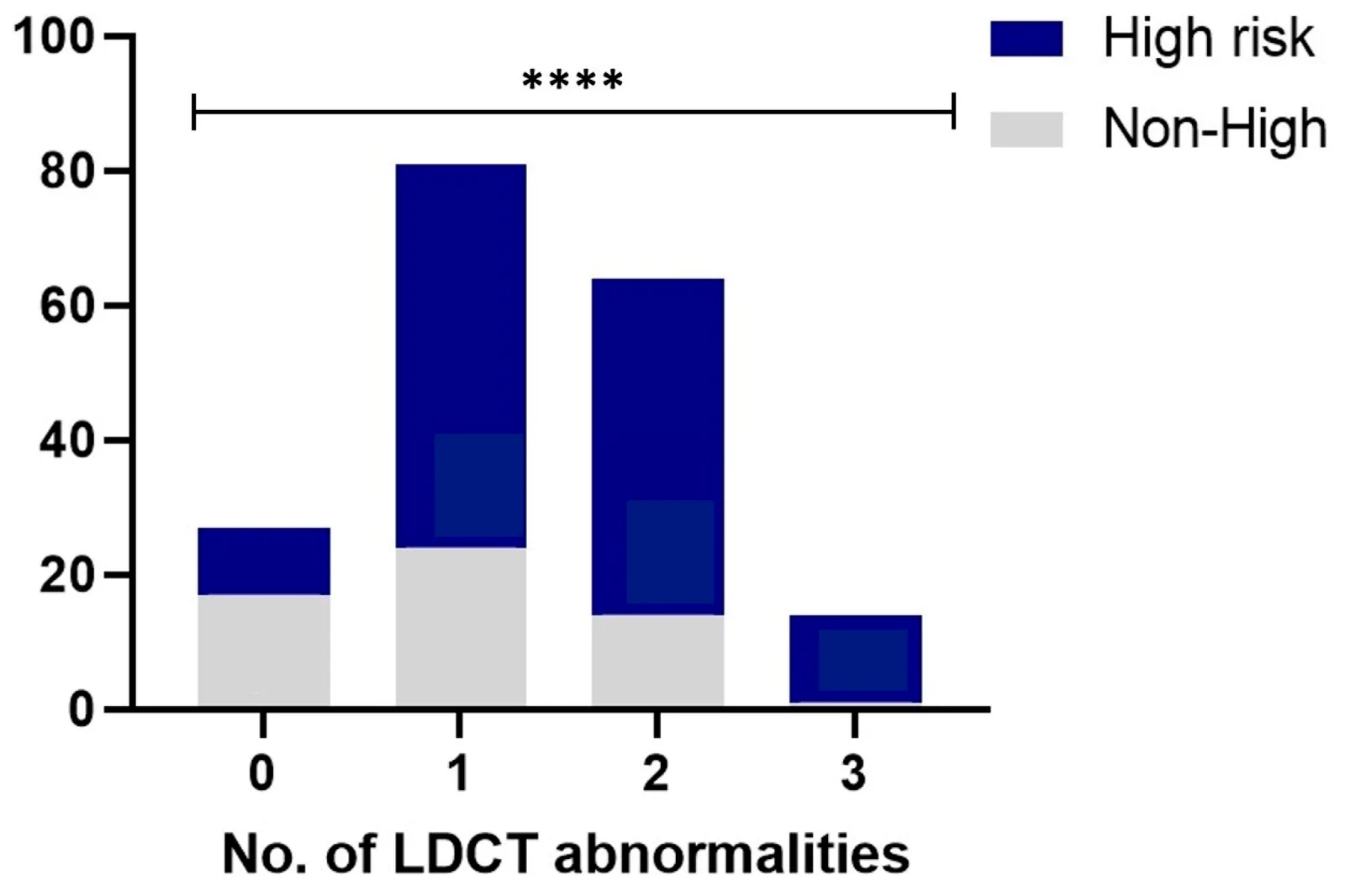
Distribution of the number of concurrent LDCT abnormalities (0–3, among nodule, emphysema, and CAC) per participant, by anamnestic risk group (non-high risk, *n* = 56; high risk, *n* = 130). High-risk participants showed a distribution shifted toward a greater number of concurrent findings (Mann–Whitney U, *p* = 0.0001). **** an extremely significant *p*-value of less than 0.0001.

The number of concurrent abnormalities (0–3 of: nodule, emphysema, CAC) per participant was also significantly higher in the high-risk group (median 2 vs. 1; Mann–Whitney U, *p* = 0.0001), with a markedly heavier burden of participants presenting 2 or 3 concurrent findings (Figure 5). Only 46/130 (35.4%) of high-risk participants with an abnormality had both a nodule and CAC concurrently, indicating that these two findings identify substantially overlapping but non-identical subsets of the screened population.

**Figure 6:**
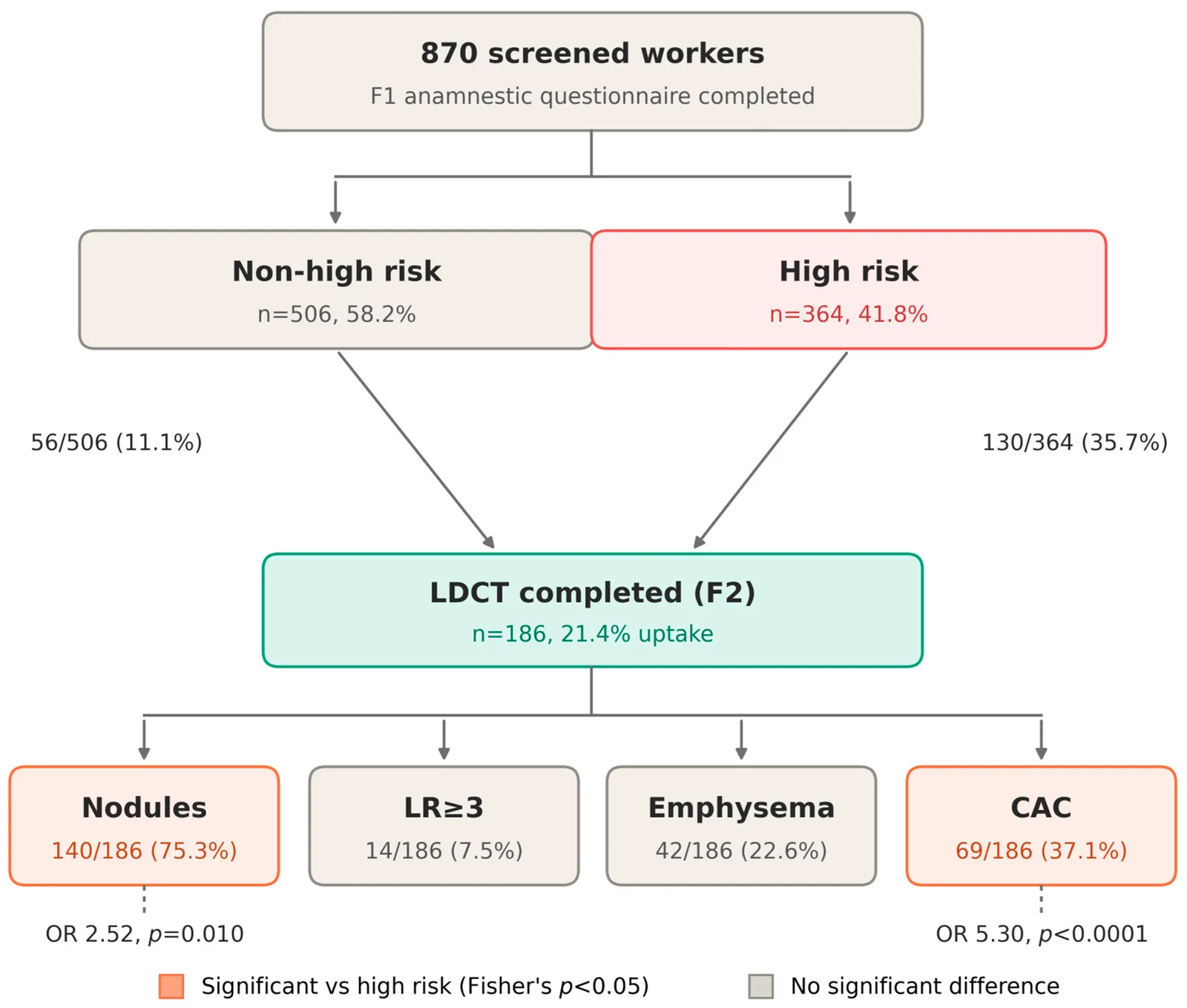
Flowchart of patients through the screening program. Of 870 screened workers, 364 (41.8%) were classified as high-risk and 506 (58.2%) as non-high-risk. LDCT uptake was voluntary and higher among high-risk workers (130/364, 35.7%) than non-high-risk workers (56/506, 11.1%), yielding 186 completed exams. Among structured LDCT findings, nodules and coronary artery calcium (CAC, shown in coral) were significantly associated with high anamnestic risk, while Lung-RADS ≥ 3 and emphysema (shown in gray) were not.

The overall patient flow, from initial screening through risk classification, LDCT uptake, and structured findings, is summarized in Figure 6.

### 3.5. Multivariable Predictors of LDCT Abnormalities

**Figure 7:**
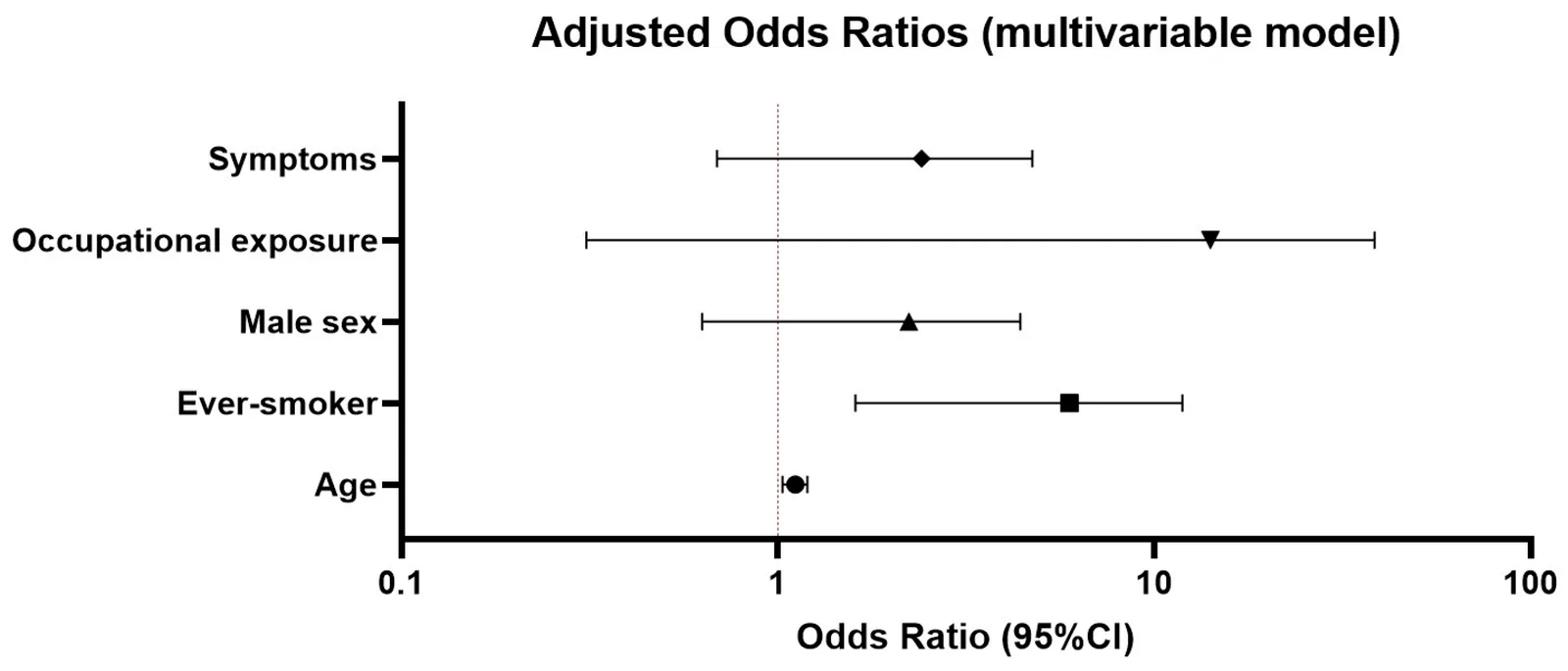
Forest plot of adjusted odds ratios (95% CI) from the multivariable logistic regression for the composite LDCT abnormality outcome.

To assess which anamnestic factors independently predicted LDCT abnormalities after mutual adjustment, a multivariable logistic regression was fitted for the composite outcome (any nodule, emphysema, or CAC), including age, ever-smoking status, sex, occupational exposure, and respiratory symptoms as covariates (complete-case analysis; no missing covariate data among the 186 F2 participants). This model should be interpreted cautiously: with 159 events and only 27 non-events among 186 participants and five predictors, the minority-class events-per-parameter (EPV) ratio was 5.4, below the conventional threshold of 10 typically recommended for stable logistic regression estimates, and occupational exposure was reported by 181/186 participants (97.3%), leaving only 5 non-exposed participants and producing a correspondingly wide confidence interval for that predictor. Variance inflation factors for all predictors were low (1.00–1.11), indicating no meaningful multicollinearity. Only age (OR 1.11 per year, 95% CI 1.03–1.20, *p* = 0.007) and ever-smoking status (OR 4.38, 95% CI 1.61–11.88, *p* = 0.004) were independently associated with the composite outcome. Sex (OR 1.66, 95% CI 0.63–4.41, *p* = 0.31), occupational exposure (OR 3.46, 95% CI 0.31–38.54, *p* = 0.31, reflecting the near-complete separation noted above), and respiratory symptoms (OR 1.80, 95% CI 0.69–4.75, *p* = 0.23) were not independently significant after adjustment (Table 6) (Figure 7). These findings indicate that, within this dataset, the discriminative value of the anamnestic risk classification for LDCT abnormalities is driven predominantly by age and smoking history rather than by the additional questionnaire domains.

Because the actionable outcomes had too few events (11–14) to support a five-predictor model, a parsimonious sensitivity model (age + ever-smoking status only) was fitted for each. Even with two predictors, EPV remained marginal (5.5–7.0). No predictor reached significance for Lung-RADS ≥ 3 (age OR 1.02, *p* = 0.74; ever-smoking OR 3.41, *p* = 0.25). For moderate-to-severe CAC, age was independently associated (OR 1.16 per year, 95% CI 1.02–1.32, *p* = 0.027) while ever-smoking status was not (OR 2.89, *p* = 0.32). These sensitivity analyses, though underpowered, are consistent with the composite-outcome regression in identifying age and, for the composite outcome, smoking, rather than the additional questionnaire domains, as the principal drivers of the observed associations; given the low event counts, they should be regarded as exploratory rather than confirmatory (Table 7).

### 3.6. Discriminative Performance of Anamnestic Risk Stratification

**Figure 8:**
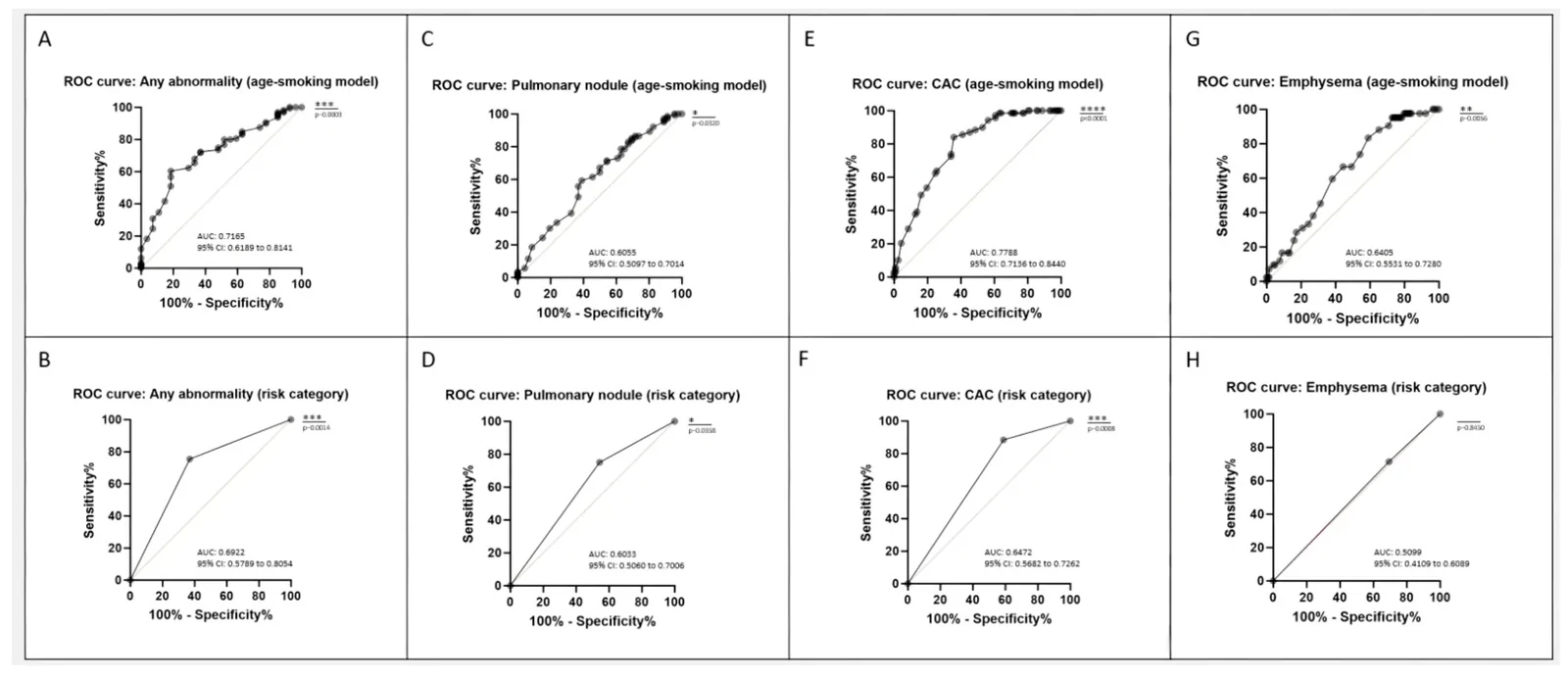
Receiver operating characteristic (ROC) curves for anamnestic predictors of LDCT findings. For each outcome, discriminative performance is shown for a continuous logistic model based on age and smoking status (left column) and for the dichotomized anamnestic risk category (non-high vs. high risk; right column). (**A**) Any abnormality, age–smoking model (AUC 0.717, 95% CI 0.619–0.814, *p* = 0.0003). (**B**) Any abnormality, risk category (AUC 0.692, 95% CI 0.579–0.805, *p* = 0.0014). (**C**) Pulmonary nodule, age–smoking model (AUC 0.606, 95% CI 0.510–0.701, *p* = 0.032). (**D**) Pulmonary nodule, risk category (AUC 0.603, 95% CI 0.506–0.701, *p* = 0.036). (**E**) Coronary artery calcium, age–smoking model (AUC 0.779, 95% CI 0.714–0.844, *p* < 0.0001). (**F**) Coronary artery calcium, risk category (AUC 0.647, 95% CI 0.568–0.726, *p* = 0.0008). (**G**) Emphysema, age–smoking model (AUC 0.641, 95% CI 0.553–0.728, *p* = 0.0056). (**H**) Emphysema, risk category (AUC 0.510, 95% CI 0.411–0.609, *p* = 0.845). AUC: area under the curve; CI: confidence interval.

To assess how well anamnestic information predicted LDCT findings, ROC analysis was performed for two classifiers: a continuous logistic model combining age and ever-smoking status, and the dichotomized anamnestic risk category (non-high vs. high risk) used for screening triage. For any abnormality (nodule, emphysema, or coronary calcium), both classifiers showed acceptable discrimination (age–smoking model: AUC 0.717, 95% CI 0.619–0.814, *p* = 0.0003; risk category: AUC 0.692, 95% CI 0.579–0.805, *p* = 0.0014), with no significant difference between them (DeLong’s test, *p* = 0.631). Discrimination of pulmonary nodules was modest and nearly identical for both classifiers (AUC 0.606 vs. 0.603; DeLong’s *p* = 0.954). In contrast, for coronary artery calcium, the continuous age–smoking model discriminated significantly better than the dichotomized risk category (AUC 0.779 vs. 0.647; DeLong’s *p* < 0.0001), and a similar, borderline pattern was observed for emphysema (AUC 0.641 vs. 0.510; DeLong’s *p* = 0.052), for which the risk category showed essentially no discriminative value. These findings indicate that dichotomization of anamnestic risk preserves most of its predictive value for pulmonary nodule detection but results in a substantial, statistically confirmed loss of information for cardiovascular and small-airway findings, which remain more closely tied to the underlying continuous variables of age and smoking history (Figure 8).

Because LDCT uptake was voluntary and, as shown in Section 3.2, was independently associated with age, smoking status, respiratory symptoms, and occupational exposure, not solely with the binary risk stratum, the ROC analyses reported above should be interpreted as characterizing the self-selected, LDCT-imaged subset rather than the full screened workforce. To characterize this selection more completely, a multivariable propensity model for LDCT uptake was fitted on the full F1 cohort, confirming that participation reflected a combination of correlated risk factors. We did not apply inverse-probability weighting to the discriminative estimates themselves, because such weighting can only adjust for the measured determinants of uptake and cannot account for unmeasured health-seeking behavior, and because the modest size of the imaged cohort would render the resulting weights unstable; the reported AUCs are therefore unweighted, and all findings in this section should be interpreted with the self-selected nature of the imaged subset in mind.

### 3.7. Clinical Pathway and Follow-Up

LDCT findings were communicated to each participant together with a Lung-RADS-based recommendation consistent with ACR guidelines [15]: annual repeat screening for Lung-RADS 1–2, short-interval follow-up LDCT at 6 months for Lung-RADS 3, and further diagnostic evaluation (including diagnostic CT, PET-CT, or tissue sampling as clinically indicated) for Lung-RADS 4A. Participants requiring bronchoscopy or biopsy were referred to a partnering oncology center (Sf. Nectarie Oncology Center); however, uptake of these referrals was not systematically tracked, as some participants declined further investigation and others chose to pursue evaluation at external institutions. No confirmed malignancy data are therefore available from this baseline screening round. Incidental findings of CAC and emphysema were documented in each participant’s structured LDCT report; referral to cardiology or pulmonology for these findings was at the discretion of the participant’s occupational physician based on the report, but adherence to such referrals was not captured in the study dataset. This study therefore reports the diagnostic yield of a single baseline screening round; longitudinal follow-up data, including nodule progression, interval cancer diagnoses, and clinical outcomes of CAC/emphysema referrals, are not yet available and will be the subject of future work.

## 4. Discussion

In this single-center occupational screening program, questionnaire-based risk stratification identified a substantial proportion of high-risk workers, driven mainly by age, smoking history, and reported respiratory symptoms. LDCT uptake rose sharply across high-risk workers (35.7% vs. 11.1%); however, because participation was voluntary and was independently associated with age, smoking, symptoms, and occupational exposure rather than with risk category alone, the F2 subsample constitutes a self-selected imaging cohort, and the association and discrimination results reported here should be interpreted as characterizing this enriched subset rather than as evidence of population-level screening performance. The multivariable uptake model (Table 4) confirms that LDCT participation reflected a combination of correlated risk factors rather than the binary risk stratum alone; however, we did not apply inverse-probability weighting to the discriminative estimates, as the modest size of the imaged cohort would render such weights unstable and weighting cannot account for unmeasured health-seeking behavior. The association and discrimination findings should therefore be interpreted with the self-selected nature of the imaged subset firmly in mind, rather than as evidence of population-level screening performance. Consistent with this triage pattern, the majority of LDCT scans were classified as Lung-RADS 1–2, with only 7.5% assigned to category ≥3. This distribution is compatible with what would be expected in a first (baseline) screening round: small, benign, or probably-benign nodules predominate, and clinically actionable findings remain uncommon in the absence of prior imaging for comparison. Reporting conventions, such as documenting multiple small solid or calcified nodules per participant, likely also shape this distribution toward the lower category. It is also worth interpreting this low positivity rate in light of the broader overdiagnosis literature in LDCT screening, which indicates that a meaningful proportion of screen-detected findings were among higher Lung-RADS categories and would never have become clinically significant, reinforcing the importance of conservative, guideline-concordant follow-up rather than aggressive early intervention [17]. This distribution is also broadly consistent with findings from other real-world European LDCT implementation cohorts outside randomized-trial settings, such as a high-risk Portuguese screening population, supporting the generalizability of our results beyond this specific occupational setting [18].

Beyond risk stratification and imaging findings, the screening encounter itself carries behavioral significance. Smoking cessation and LDCT screening appear to act synergistically rather than redundantly in reducing lung cancer mortality. In a secondary analysis of NLST participants, seven years of sustained smoking abstinence was associated with a mortality reduction of similar magnitude to LDCT screening itself, and the combination of long-term abstinence with screening produced the largest reduction observed [19]. A concern sometimes raised is that screening might give smokers a false sense of reassurance (“permission to keep smoking”); however, a systematic review conducted for the U.S. Preventive Services Task Force found that undergoing LDCT screening itself does not measurably change smoking behavior, while a positive or concerning scan result is associated with increased abstinence, suggesting the screening encounter can function as a genuine teachable moment rather than a source of false reassurance [20]. This has direct relevance to eligibility considerations in our cohort: the American Cancer Society recommends annual LDCT for asymptomatic adults aged 50–80 with a ≥20 pack-year smoking history who currently or formerly smoked [21]. Lung cancer incidence has been declining in the United States since the early 1990s, and mortality declines have accelerated further in recent years for both sexes, a trend attributed to tobacco control efforts, historical differences in smoking uptake and cessation between men and women, and, more recently, improvements in treatment and early detection [21]. Similar patterns of persistent disease burden alongside disparities in early-detection access have been documented across Europe, underscoring the relevance of workplace-based screening initiatives in reaching populations that may not otherwise engage with organized national screening pathways [22]. Randomized trial evidence, together with diagnostic accuracy data showing high sensitivity and acceptable specificity for early lung cancer detection in high-risk individuals, underpins these recommendations [21].

Beyond its role in lung cancer detection, LDCT in this cohort surfaced findings with direct relevance to chronic disease prevention. Nearly one-quarter of examined workers had emphysema, a finding that supports pairing LDCT-based screening with spirometric evaluation and targeted smoking-cessation support, since emphysema on LDCT is not only a marker of airflow obstruction but has also been shown, independent of smoking history, to predict lung cancer incidence, lung cancer mortality, and all-cause mortality [23]. This reflects a broader, increasingly recognized role for LDCT lung cancer screening as a platform for detecting smoking-related comorbidities beyond malignancy [24]. Coronary artery calcium (CAC) was similarly frequent (37.1%) and was closely linked to higher questionnaire-derived risk. This finding is consistent with a growing evidence base establishing CAC identified on lung-cancer-screening CT as a meaningful, low-cost predictor of cardiovascular events, without the need for a dedicated cardiac-gated scan [25]. Taken together, these findings support using the LDCT encounter as an opportunity—a “teachable moment”, to engage workers, particularly older smokers, in cardiovascular risk discussion and follow-up, alongside the primary lung cancer screening objective.

It is worth noting that the present program occupies a middle ground between two distinct but overlapping approaches. Conventional lung cancer screening, as established by the NLST and NELSON trials, targets a narrowly defined high-risk population (typically by age and pack-year thresholds) with the specific objective of reducing lung cancer mortality through early detection [6,7]. Occupational respiratory health surveillance, by contrast, aims broadly to detect and monitor work-related respiratory and cardiopulmonary disease in an exposed workforce, regardless of cancer risk specifically. The present two-step strategy combines elements of both: it uses anamnestic risk factors, including but not limited to smoking, to prioritize workers for LDCT, while the imaging itself captures not only oncologic findings (Lung-RADS) but also emphysema, coronary calcium, and other incidental pathology relevant to occupational and general health. This dual objective should inform how the study’s yield is interpreted: the high prevalence of incidental findings and the relatively low rate of actionable Lung-RADS categories are expected when screening serves a surveillance as well as a cancer-detection function, and the value of the program should be assessed against both objectives rather than against oncologic yield alone.

Free-text incidental findings suggested a non-trivial burden of fibrotic scarring and pleural changes. In coal-exposed workers specifically, coal mine dust and cigarette smoking have been shown to interact, producing non-pneumoconiotic fibrosis detectable on both imaging and histology beyond what either exposure produces alone [26]; however, this literature derives predominantly from coal-mining (extraction) cohorts with characterized cumulative dust exposure, which differs materially from the combustion-related fly-ash exposure relevant to coal-fired power generation, and the two settings should not be assumed to carry equivalent respiratory risk. In the present study, occupational exposure was captured as a single yes/no questionnaire item, without quantitative information on job category, task, duration of employment, cumulative exposure, or use of personal protective equipment, and was reported by nearly all participants (94.5% in F1), leaving little discriminative information within this variable and precluding any dose–response or exposure-stratified analysis. Consequently, the associations reported in this study cannot be used to establish a causal contribution of coal-fired power plant exposure specifically to the imaging findings observed; they are equally compatible with age- and smoking-driven pathology unrelated to the occupational setting. Future iterations of this screening program should incorporate standardized occupational-exposure assessment, including job-exposure matrices, duration and cumulative exposure metrics, and protective equipment use, alongside standardized occupational-imaging frameworks and paired spirometry, to permit causal inference regarding this specific occupational setting.

From an operational standpoint, the two-step strategy offers several practical advantages: it is scalable and cost-efficient at the initial triage stage consistent with broader systematic-review evidence that risk-based pre-screening improves the budget impact and cost-effectiveness profile of LDCT programs compared with unrestricted eligibility [27] aligns with established LDCT eligibility principles, identifies symptomatic workers who warrant medical evaluation independent of the oncologic screening pathway, and produces a unified, linked dataset that supports integrated follow-up planning. This dual-exposure, framing occupational particulate exposure combined with smoking, mirrors emerging cost-effectiveness evidence from other occupationally exposed European populations, where combined asbestos and smoking exposure has similarly been used to justify targeted LDCT screening programs [28]. Nonetheless, selection bias is inherent to this design: LDCT utilization was shaped jointly by risk stratification and voluntary participation, which limits direct comparison of raw prevalence rates across risk groups, as noted above.

A limitation acknowledged by this study is the absence of follow-up outcome data beyond the baseline screening round. Although a clinical referral pathway was established for participants with actionable Lung-RADS findings, systematic tracking of referral uptake, diagnostic confirmation, and downstream clinical outcomes was not implemented, and some participants declined further evaluation or pursued it independently at other institutions. Consequently, the present study reports screening yield and risk-group associations at baseline, but cannot yet demonstrate whether the proposed two-step strategy leads to earlier diagnosis, treatment, or improved clinical outcomes, a distinction that is essential for interpreting claims about the value of any screening program.

The association between high anamnestic risk and greater nodule prevalence likely reflects several overlapping factors: the age-related rise in incidental benign nodules and smoking-related pulmonary changes; a specific occupational contribution cannot be established from the present exposure data. The multivariable regression results support this interpretation directly: after mutual adjustment, only age and ever-smoking status remained independent predictors of the composite abnormality outcome, while occupational exposure and respiratory symptoms did not, suggesting that age and smoking history are the principal drivers of the observed associations rather than the full multi-domain questionnaire or occupational exposure specifically, though this model was likely underpowered and this null finding for occupational exposure should not be over-interpreted given the extremely wide confidence interval. Despite the overall association, the small proportion of Lung-RADS ≥3 findings suggests that most detected nodules carried low clinical concern at baseline. Ongoing longitudinal follow-up will be necessary to assess nodule progression, potential reclassification, and the resulting diagnostic yield. This is particularly important given evidence that adherence to subsequent screening rounds after a negative baseline result varies substantially depending on program structure, with centralized, coordinated screening pathways similar to the unified dataset approach used here are associated with better long-term adherence than decentralized models [29].

### Strengths and Limitations

The strengths of this study include the large size of the screened population assessed with a standardized questionnaire, deterministic linkage between questionnaire and imaging data, the use of structured LDCT reporting incorporating Lung-RADS v2022 alongside cardiovascular co-reporting, and the discriminative-performance and multivariable analyses that directly quantify which anamnestic components carry independent predictive value. Several limitations should be considered. First, LDCT uptake was voluntary and differed substantially by risk group; although a multivariable propensity model was fitted to characterize this selection (Table 4), inverse-probability weighting was not applied to the discriminative estimates because the modest cohort size would render the weights unstable and weighting cannot account for unmeasured health-seeking behavior. Residual confounding therefore cannot be excluded, and all association and discrimination findings should be understood as describing this self-selected imaging subset rather than the full screened workforce. Second, the primary composite outcome combines findings of markedly different clinical significance; when restricted to actionable thresholds, no outcome remained significantly associated with anamnestic risk, and this distinction should guide interpretation of the study’s practical implications. Third, occupational exposure was assessed with a single non-quantitative item reported by nearly all participants, precluding any dose–response assessment and limiting causal inference specific to the coal-fired power plant setting, which should be clearly distinguished from the more extensively characterized coal-mining exposure literature. Fourth, a clinical referral pathway was established for participants with actionable Lung-RADS findings, follow-up outcomes (referral uptake, diagnostic confirmation, interval malignancy, and clinical outcomes of CAC/emphysema referrals) were not systematically tracked, and this study therefore reports baseline screening yield only; the absence of longitudinal outcome data limits the ability to assess the clinical effectiveness of the proposed screening strategy. Fifth, the cross-sectional, single-round design precludes assessment of nodule progression, reclassification rates, or adherence to subsequent screening rounds. Sixth, the multivariable regression for the composite outcome had a minority-class events-per-parameter ratio of 5.4, below conventional recommendations, and the corresponding sensitivity models for actionable outcomes were similarly underpowered; these analyses should be regarded as exploratory. Seventh, all LDCT examinations were interpreted by a single radiologist without independent second-reader verification, which may have introduced inter-observer variability not captured in this analysis, particularly for borderline Lung-RADS categorization and subjective assessments such as emphysema severity and visual coronary calcium scoring. Additional limitations include missing data for some questionnaire fields, potential inaccuracies in anthropometric self-report, the absence of standardized quantitative smoking exposure (pack-years), lack of spirometric confirmation of airflow obstruction, and reliance on free-text reporting for non-nodular incidental findings.

## 5. Conclusions

Among workers at a coal-fired power plant, integrating questionnaire-based risk classification with voluntary LDCT imaging, enriched the imaged subset with a higher overall burden of incidental cardiopulmonary findings and with smoking- and age-related risk factors. However, when restricted to clinically actionable outcomes, Lung-RADS ≥3, moderate-to-severe CAC, and moderate-to-severe emphysema, no significant association with anamnestic risk was observed. Multivariable and discriminative-performance analyses indicate that age and smoking history, rather than the full multi-domain questionnaire, are the principal drivers of this association. Given the voluntary and differential nature of LDCT uptake across risk strata, these findings are best regarded as hypothesis-generating: they support the feasibility of an integrated occupational health model incorporating lung cancer screening, COPD assessment, smoking-cessation intervention, and cardiovascular risk management, but do not by themselves establish that anamnestic risk stratification reliably identifies workers who will derive clinical benefit from LDCT, nor that the referral pathway established for actionable findings resulted in confirmed diagnoses or improved outcomes. Future research should incorporate longitudinal imaging follow-up, objective pulmonary function testing, and quantitative occupational exposure assessment in a less selected screening population to confirm these findings and better disentangle the relative contributions of occupational exposure and lifestyle factors to the respiratory disease burden observed.

## Figures and Tables

**Table 1:** Baseline characteristics of screened workers (F1) and the LDCT subset (F2).

Characteristic	F1: All Screened (*n* = 870)	F2: LDCT Subset (N = 186)
Age, years (mean ± SD)	49.3 ± 7.5	51.6 ± 5.9
Male sex	628 (72.2%)	132 (71.0%)
Current smoker	226 (26.0%)	80 (43.0%)
Former smoker	202 (23.2%)	70 (37.6%)
Never a smoker	409 (47.0%)	31 (16.7%)
Passive smoke exposure	33 (3.8%)	5 (2.7%)
Occupational exposure reported	810/857 (94.5%) *	181/186 (97.3%) *
Respiratory symptoms reported	189/861 (22.0%) *	67/183 (36.6%) *

* Percentages calculated among respondents with non-missing data for the variable.

**Table 2:** Multivariable logistic model of LDCT uptake in the full screened cohort (*n* = 870).

Predictor	Adjusted OR	95% CI	*p*-Value
Age (per year)	1.06	1.03–1.09	<0.001
Ever-smoker	6.51	4.28–9.89	<0.001
Respiratory symptoms	1.98	1.34–2.91	<0.001
Occupational exposure	3.66	1.38–9.73	0.009

**Table 3:** Key LDCT findings (N = 186).

Finding	*n* (%)
Any pulmonary nodule	140 (75.3%)
Lung-RADS 0	8 (4.3%)
Lung-RADS 1	67 (36.0%)
Lung-RADS 2	97 (52.2%)
Lung-RADS 3	10 (5.4%)
Lung-RADS 4A	4 (2.2%)
Lung-RADS ≥ 3	14 (7.5%)
Emphysema (any) Emphysema (moderate/confluent)	42 (22.6%) 4 (2.2%)
Coronary calcium (any) Coronary calcium (moderate-severe, ≥1/3)	69 (37.1%) 11 (5.9%)
Fibrotic changes (free text)	18 (9.7%)
Pleural changes (free text)	10 (5.4%)
Airway mucus/bronchitis features (free text)	17 (9.1%)
Infectious/inflammatory features (free text)	12 (6.5%)

**Table 4:** Association between high anamnestic risk and selected LDCT outcomes.

Outcome	Non-High Risk (*n* = 56)	High Risk (*n* = 130)	OR (Fisher)	95% CI	*p*-Value
Lung-RADS ≥ 3	4 (7.1%)	10 (7.7%)	1.08	0.32–3.61	1.000
Any nodule	35 (62.5%)	105 (80.8%)	2.52	1.26–5.05	0.010
Emphysema (any)	12 (21.4%)	30 (23.1%)	1.10	0.52–2.35	0.851
Coronary calcium (any)	8 (14.3%)	61 (46.9%)	5.30	2.33–12.09	<0.0001

**Table 5:** Association between high anamnestic risk and clinically actionable LDCT outcomes.

Actionable Outcome	Non-High Risk (*n* = 56)	High Risk (*n* = 130)	OR	*p*-Value
Lung-RADS ≥ 3	4 (7.1%)	10 (7.7%)	1.08	1.000
CAC, moderate-severe (≥1/3)	1 (1.8%)	10 (7.7%)	4.58	0.178
Emphysema, moderate-severe	1 (1.8%)	3 (2.3%)	1.30	1.000

**Table 6:** Multivariable logistic regression for the composite LDCT abnormality outcome (any nodule, emphysema, or CAC), with model diagnostics.

Predictor	Adjusted OR	95% CI	95% CI
Age (per year)	1.11	1.03–1.20	0.007
Ever-smoker	4.38	1.61–11.88	0.004
Male sex Occupational exposure Respiratory symptoms	1.66 3.46 1.80	0.63–11.88 0.31–38.54 0.69–4.75	0.306 0.313 0.232

Model diagnostics: complete-case analysis (*n* = 186, no missing covariate data); 159 events, 27 non-events; minority-class events-per-parameter (EPV) = 5.4 (below the conventional threshold of 10). Variance inflation factors: age 1.04, ever-smoker 1.11, male sex 1.11, occupational exposure 1.03, respiratory symptoms 1.00 (all <2, indicating no meaningful multicollinearity). The wide confidence interval for occupational exposure reflects near-complete separation (only 5/186 participants occupationally unexposed). CI, confidence interval; OR, odds ratio; CAC, coronary artery calcium.

**Table 7:** Parsimonious sensitivity models (age + ever-smoking status) for clinically actionable outcomes.

Outcome	Predictor	Adjusted OR	95% CI	*p*-Value
Lung-RADS ≥ 3 (14 events)	Age (per year)	1.02	-	0.74
	Ever-smoker	3.41	-	0.25
CAC moderate-severe (11 events)	Age (per year) Ever-smoker	1.16 2.89	1.20–1.32 -	0.027 0.32

Events-per-parameter remained marginal (5.5–7.0). Moderate-to-severe emphysema (4 events) had too few events for a stable model and was not fitted. CI, confidence interval; OR, odds ratio; CAC, coronary artery calcium.

## Data Availability

The original contributions presented in this study are included in the article/Supplementary Material. Further inquiries can be directed to the corresponding author.

## Supplementary Materials

The following supporting information can be downloaded at: https://www.mdpi.com/article/10.3390/diagnostics16193202/s1, Supplementary Material S1: Derivation of the binary anamnestic risk classification used throughout this manuscript.

## Abbreviations

The following abbreviations are used in this manuscript:

LDCT	Low-dose computed tomography
CAC	Coronary artery calcium
COPD	Chronic obstructive pulmonary disease
EPV	Events-per-parameter
NLST	National Lung Screening Trial
NELSON	Dutch–Belgian Randomized Lung Cancer Screening Trial
Lung-RADS	Lung CT Screening Reporting and Data System
OR	Odds ratio
CI	Confidence interval
AUC	Area under the (ROC) curve
ROC	Receiver operating characteristic
IQR	Interquartile range
CTDIvol	Volume computed tomography dose index
