# Supplementary material for: Two-Phase Occupational Screening in Coal-Fired Power Plant Workers: Anamnestic Risk Stratification and Targeted Low-Dose CT Findings
Source: Diagnostics (Basel). 2026 Oct 2;16(19):3202. doi: 10.3390/diagnostics16193202 (PMC13664029; doi:10.3390/diagnostics16193202)
Supplement: Supplementary file 1 [file diagnostics-16-03202-s001.zip › diagnostics-4556105-supplementary.pdf]

Supplementary Material S1

HighRisk was applied identically to all 870 F1 participants:

EligibilityRuleRisk = High if RespiratorySymptoms_Flag = 1 OR AgeSmoker_Flag = 1

EligibilityRuleRisk = Non-high otherwise

where:

RespiratorySymptoms_Flag = 1 if questionnaire item "respiratory symptoms" = Yes, else 0

AgeSmoker_Flag = 1 if age ≥ 50 years AND smoking status ∈ {current smoker, former smoker}, else 0
